# An Evaluation of Antimicrobial, Anticancer, Anti-Inflammatory and Antioxidant Activities of Silver Nanoparticles Synthesized from Leaf Extract of *Madhuca longifolia* Utilizing Quantitative and Qualitative Methods

**DOI:** 10.3390/molecules27196404

**Published:** 2022-09-28

**Authors:** Pooja Salve, Aruna Vinchurkar, Rajesh Raut, Ramesh Chondekar, Jaya Lakkakula, Arpita Roy, Md. Jamal Hossain, Saad Alghamdi, Mazen Almehmadi, Osama Abdulaziz, Mamdouh Allahyani, Anas S. Dablool, Md. Moklesur Rahman Sarker, Mohd Fahami Nur Azlina

**Affiliations:** 1 Department of Biophysics, Government Institute of Science, Aurangabad 431004, India; 2 Department of Botany, The Institute of Science, 15 Madame Cama Road, Mumbai 400032, India; 3 Department of Zoology, Dr. Babasaheb Ambedkar Marathwada University, Aurangabad 431004, India; 4 Amity Institute of Biotechnology, Amity University Maharashtra, Mumbai-Pune Expressway, Mumbai 410206, India; 5 Department of Biotechnology, School of Engineering & Technology, Sharda University, Greater Noida 201310, India; 6 Department of Pharmacy, State University of Bangladesh, 77 Satmasjid Road, Dhanmondi, Dhaka 1205, Bangladesh; 7 Laboratory Medicine Department, Faculty of Applied Medical Sciences, Umm Al-Qura University, Makkah 24382, Saudi Arabia; 8 Department of Clinical Laboratory Sciences, College of Applied Medical Sciences, Taif University, Taif 21944, Saudi Arabia; 9 Department of Public Health, Health Sciences College at Al-Leith, Umm Al-Qura University, Makkah 24382, Saudi Arabia; 10 Department of Pharmacology, Faculty of Medicine, University Kebangsaan Malaysia, Jalan Yaacob Latif, Kuala Lumpur 56000, Malaysia

**Keywords:** silver nanoparticle, antibacterial, anti-inflammation, *Madhuca longifolia*, antioxidation, anticancer

## Abstract

In the current decade, nanoparticles are synthesized using solvents that are environmentally friendly. A number of nanoparticles have been synthesized at room temperature using water as a solvent, such as gold (Au) and silver (Ag) nanoparticles. As part of nanotechnology, nanoparticles are synthesized through biological processes. Biological methods are the preferred method for the synthesis of inorganic nanoparticles (AgNPs) as a result of their simple and non-hazardous nature. Nanoparticles of silver are used in a variety of applications, including catalysts, spectrally selective coatings for solar absorption, optical objectives, pharmaceutical constituents, and chemical and biological sensing. Antimicrobial agents are among the top uses of silver nanoparticles. In the current study, silver nanoparticles were biologically manufactured through Madhuca longifolia, and their antibacterial activity against pathogenic microorganisms, anticancer, anti-inflammatory, and antioxidant activities were assessed. UV-Vis spectroscopy, XRD (X-ray diffraction), transmission electron microscopy, Zeta Potential, and FTIR were used to characterize silver nanoparticles. The current work describes a cheap and environmentally friendly method to synthesize silver nanoparticles from silver nitrate solution by using plant crude extract as a reducing agent.

## 1. Introduction

Modern material science is experiencing a boom in nanotechnology, and the synthesis of nanoparticles has become increasingly important because of its applications in a wide range of fields. The science of nanotechnology, dealing with materials at the nanometer scale, can be applied to all fields of science, including chemistry, biology, physics, materials science, and engineering [1].

Nanoparticles are being synthesized using plants due to their simplicity and eco-friendliness. *Madhuca longifolia,* also known as Mahua, is a member of the Sapotaceae family. Mahua is a deciduous tree that grows in shady areas. Various parts of the plant are used in the ancient Indian medicine system, including the seeds, flowers, roots, bark, and whole young plants [2]. Parts of the plants are also used for treating diseases including cholera, paralysis, snake bites, debility, tonsillitis, influenza, piles, arthritic pain, helminthiasis, headaches, low semen count, flatulence, rheumatoid arthritis, tuberculosis, and infection [3]. The bioactive molecules present in *Madhuca longifolia* plant parts are carbohydrates, flavonoids, glycosides, triterpenoids, phenolic compounds and tannins, which are responsible for its ability to protect against pathogens and have been a rich source of medicinal compounds [4,5]. Quercetin is a flavone that occurs in the leaves of *Madhuca longifolia*; it has beneficial antibacterial, antiviral, anti-carcinogenic, and anti-inflammatory effects as well as being a potential therapeutic drug for treating psoriasis [6]. There are important secondary metabolites in *Madhuca longifolia* seeds that can be used to prepare drugs to treat skin diseases [7]. Madhuca seed oil and flower oil are rich in medicinal values and can be used to treat ailments like pain relief, oleation, nausea, and urinary ailments. Ayurvedic medicine holds *Madhuca longifolia* in high regard as a universal panacea. It is used as an antidote for snakebites [8]. The extract of *Madhuca longifolia* is regarded as a potential antimicrobial agent [9]. Phytochemical study of absolute ethanol extract of the whole plant has shown that it contains flavonoids, glycosides, carbohydrates, and tannins responsible for pharmacological activity having anti-inflammatory, antioxidant, chemoprotective, anti-diabetic, antianxiety, and antidepressant effects [10].

Nanomaterials have very unique properties compared to their bulk counterparts. In addition, they have a very large surface area which typically results in their high reactivity [11]. The synthesis of silver nanoparticles has received considerable attention due to their potential application in reduced oxidative stress, diabetes, liver diseases, inflammatory, cancer and pharmacological application, dairy products [12], and other industrial uses [13], In the present study, we have used *Madhuca longifolia* (vernacular name—mahua or mohe) leaves for the synthesis of silver nanoparticles. The synthesized nanoparticles were characterized for their size, shape, crystallinity, and surface morphology and evaluated for antimicrobial activity against human pathogenic microorganisms, antioxidation, anti-inflammatory activity, and anticancer activity.

## 2. Materials and Methods

### 2.1. Materials

Silver nitrate (Sigma Aldrich, Waltham, MA, USA) was used as received. Other chemicals such as 2,2,diphenyl-1-picrylhydrazyl (DPPH) (Sigma Aldrich), ascorbic acid, and ready-to-use bacterial media (Muller Hilton agar, Hi-Media) were used without any further processing. Double distilled water was used in all experiments. Whatman filter paper number 1 (11 µm) was used to filter the plant extract.

### 2.2. Methods

#### 2.2.1. Preparation of Leaf Extract

The mature, undamaged, and disease-free leaves of *Madhuca longifolia* were collected from Chauka ghat, the north region of district Aurangabad, Maharashtra, India. Taxonomic identification was made with the help of the herbarium obtained from the Department of Botany at Dr. Babasaheb Ambedkar Marathwada University, Aurangabad, India. Accession No: 0709. The plant leaves were washed thoroughly with double distilled water wrapped in newspaper and dried under shade. The dried leaves were segregated, pulverized by a mechanical grinder and sieved using 1.5 mm mesh, and further stored in a plastic box at room temperature. The crude extract of the plant material was prepared as described by [14] with some modifications. Half a gram of plant powder was mixed with 100 mL of double-distilled water followed by mild sonication (50 Hz frequencies for 1 min. with the pulse rate of 10:2 using bath sonicator Ultrasonic cleaner, K615HTDP). The crude extract was then filtered using Whatman filter paper number 1 to get a clear solution, which was used as the stock solution.

#### 2.2.2. Synthesis of Silver Nanoparticles

Under room temperature, 10 mL of a leaf extract stock solution was added to 90 mL of silver nitrate solution at 1 mM. After mixing thoroughly, the solution was exposed to sunlight for 5 min. The solution changed from yellow to brown. The colored solution was centrifuged at 10,000 rpm for 15 min to obtain pellets [15]. To produce nanoparticles free of any biological material present in synthesized silver nanoparticles, the pellets were washed with absolute ethanol and dispersed in sterile distilled water [16].

#### 2.2.3. Characterization of Silver Nanoparticles

Spectroscopic studies (UV-visible spectrophotometer Systronic 2203) confirmed the formation of silver nanoparticles. An FTIR analysis was performed of the reaction mixture and plant extract after exposure to sunlight using FTIR (BRUKER Alpha) to identify possible biomolecules involved in the reduction of silver ions. This was described by using FTIR (BRUKER Alpha) in the spectral range of 400–4000 cm^−1^. With an X-ray diffractometer BRUKER D8 Advance operating at 40 kv and 40 mA, Cukα radian at a 2θangle was used to determine the crystalline structures of the silver nanoparticles. 

A TEM (PHILIPS CM 200) was used to determine the morphology and the mean particle size of the silver nanoparticles. A TEM analysis was performed according to [17].

#### 2.2.4. Antibacterial Studies

Using a paper disk diffusion method, *Madhuca longifolia* leaf extract and silver nanoparticles synthesized from the leaves extract of *Madhuca longifolia* were tested against *E. coli* gram-negative bacteria [18]. As the growth medium for bacterial cultures, Mueller Hinton Agar media was prepared, sterilized, and poured into sterilized Petri plates. The sterilized media was covered and allowed to solidify. A sterile cotton swab was used to seed the plates; meanwhile, the sterilized 6 mm paper disks were soaked in the silver nanoparticle solution, varying its concentration, and allowed to dry at room temperature. Once the paper disk was dried, it was placed on plates and incubated for 24 h at 37 °C to measure inhibition zones. As an antibacterial standard, Ciprofloxacin 5 μg/disc was used. 

#### 2.2.5. Anti-Inflammatory Studies

Silver nanoparticles synthesized were tested for their anti-inflammatory properties by inhibiting protein denaturation and using the HRBC method. For inhibition of protein denaturation, 1% aqueous solutions of BSA (bovine serum albumin) were diluted with different concentrations of silver nanoparticles synthesized from *Madhuca longifolia*. The reaction mixture was carried out at the pH of 7. After incubating at 37 °C for 20 min, the mixtures were heated again to 51 °C for 20 min. Once the samples were cooled, spectrophotometric measurements were performed at 660 nm. A standard drug was used, diclofenac sodium. Three replicates of each experiment were performed [19].

Percent inhibition of protein denaturation was calculated as follows:

Percent Inhibition = 100 − (O. D. of test sample − O. D. of control sample) × 100/O.D. of control.

The HRBC method has been used to study heat-induced hemolysis and hypotonicity-induced hemolysis. Before conducting the experiments, the membranes were stabilized. Blood was collected from a healthy human volunteer who had not taken NSAIDs (Non-Steroidal Anti-Inflammatory Drugs) for 2 weeks before the experiment to stabilize the membrane. Centrifuged for 10 min at 3000 rpm and washed three times with normal saline. The blood volume was measured and reconstituted as 10% *v*/*v* suspension with normal saline. The method was developed by [20] and modified by [21].

During heat-induced hemolysis, the reaction mixture (2 mL) included a 1 mL test sample, control and a standard drug (100–500 g/mL AgNPs, saline, and aspirin, respectively), and a 1 mL suspension of 10% RBCs that were mixed and centrifuged. After centrifugation, the reaction mixture was incubated in a water bath at 56 °C for 30 min. The tubes were cooled under running tap water at the end of the incubation. The reaction mixture was centrifuged again at 2500 rpm for 5 min, and the absorbance of the supernatants was measured at 560 nm. All samples were tested in triplicate [22,23].

The Percentage inhibition of Hemolysis was calculated as follows:

Percentage inhibition = (Abs control − Abs sample) × 100/Abs control)

##### Hypotonicity-Induced Hemolysis

In this test, Madhuca longifolia AgNps samples were suspended in distilled water (hypotonic solution). Each centrifuge tube was filled with hypotonic solution (5 mL) containing graded doses of the extract (100, 200, 300, 400, and 500 μg/mL). Additionally, duplicate pairs (per dose) of isotonic solution (5 mL) containing graded doses of the extracts (100–500 μg/ mL) were placed in the centrifuge tubes. The control tubes contained 5 mL of the vehicle (distilled water) and 5 mL of sodium Diclofenac 200 mg/mL. The erythrocyte suspension (0.1 mL) was added to each tube and mixed gently. After incubation for 1 h at room temperature (37 °C), the mixtures were centrifuged for 3 min at 1300 g. To determine the hemoglobin concentration in the supernatant, using a spectrophotometer (Chemito UV 2100), the absorbance (OD) was measured at 560 nm. The hemolysis percentage was calculated by considering the hemolysis produced in distilled water as 100%. As a result, the percent inhibition of hemolysis by the extract was calculated.

Percentage protection = 100 − (OD sample/OD control) × 100 [20].

#### 2.2.6. Antioxidation Studies

The DPPH dot blot method was used in the study of antioxidation as described by [24]. To begin, 3μl of synthesized silver nanoparticles were loaded on silica gel on a glass plate. After allowing the spot to dry, it was placed in a coupling jar containing 0.4 mM DPPH for ten seconds. Free radical scavenging was indicated by a white spot on a light purple background.

In addition to spectrophotometric studies, the scavenging activity of free radicals was also studied [25]. 24 mg of DPPH were dissolved in 100 mL of methanol to prepare the stock solution, which was stored at 20 °C until needed. With the spectrophotometer, we measured the absorbance of the DPPH working solution at 517 nm as 0.98 ± 0.02. A 3 mL aliquot of this solution was mixed with 100 μl of the silver nanoparticles at various concentrations (100–500 μg/mL). After shaking well, the reaction mixture was incubated in the dark at room temperature for 15 min. After that, the absorbance was measured at 517 nm. We used ascorbic acid as a reference standard and a reaction without a sample as a control.

Based on the percentage of DPPH radicals scavenged, the following equation can estimate the scavenging activity:

Scavenging effect % = [(control absorbance-sample absorbance)/(control absorbance)] × 100

#### 2.2.7. Anticancer Studies

The anticancer properties of silver nanoparticles prepared using M longifolia extract in different concentrations (10, 20, 40, 80 μg/mL) have been examined using the SiHa cervical cancer cell line as well as the MDA-MB-231 breast cancer cell line. ACTREC (Advanced Centre for Treatment Research and Education in Cancer) in Mumbai, India, conducted anticancer studies. An SRB assay was used to test cell viability. Each concentration of silver nanoparticles was tested in triplicate. Growth curves were plotted against silver nanoparticle concentrations of μg/mL and percent control growth with adriamycin as a positive control.

Experimental procedure or SRB assay

Human breast cancer cell line MDA-MB-231 and human cervical cancer cell line SiHa were grown in RPMI 1640 medium containing 10% Fetal bovine serum and 2 mM L-glutamine. A 96-well microtiter plate was used for screening silver nanoparticles. Before adding silver nanoparticles of varying concentrations to the plates, the microtiter plates were incubated at 37 °C, 5% CO_2_, 95% air, and 100% relative humidity for 24 h after cell inoculation. In situ fixation of a single plate of each cell line with TCA was performed after 24 h of incubation to give a representation of the cell population before silver nanoparticles were added. To achieve concentrations of 10, 20, 40, and 80 nanoparticles μ/mL silver nanoparticles, 10 μl aliquots of silver nanoparticles were added to microtiter wells already containing 90 μl of the medium. The assay was terminated with the addition of cold TCA after 48 h of incubation after the addition of silver nanoparticles. The cells were fixed by 10% TCA. A 60-min incubation at 40 C was followed by 5 washes with distilled water and air drying, followed by staining with Sulforhodamine B (SRB) solution (50 mL) at 0.4% (*w*/*v*) in 1% acetic acid. After 20 min at room temperature, the plates were incubated. After removing the excess stain with 1% acetic acid, the plate was air dried and read on an Elisa plate reader at 540 nm with a reference wavelength of 690 nm [26]. The percent growth was calculated by comparing the average absorbance of the test well with the average absorbance of the control wells × 100. As a result of the six absorbance measurements (time zero (Tz), control growth (C)), and test growth in the presence of silver nanoparticles at four concentration levels (Ti), growth inhibition was 50%.

GI50 = [(Ti-Tz)/(C-Tz)] × 100

GI50 is the concentration of silver nanoparticles that results in a 50% reduction in the increases in protein in control cells (as measured by SRB staining) during the incubation with silver nanoparticles. The silver nanoparticles concentration resulting in total growth inhibition (TGI) was calculated from Ti = Tz. LC50 is the concentration of silver nanoparticles that causes a 50% reduction in protein measured at the end of the treatment compared to the beginning. There is a net loss of 50% cells following this treatment, calculated as [(Ti-Tz)/Tz] × 100 = −50 [27].

Statistical Analysis

These analyses were done at least in triplicate and the results are presented as averages. ANOVA was used to analyze differences between groups, and Tukey’s multiple comparison test was used to determine significance.

## 3. Result and Discussion

### 3.1. UV-Vis (Ultraviolet-Visible Spectroscopy Analysis)

**Figure 1:**
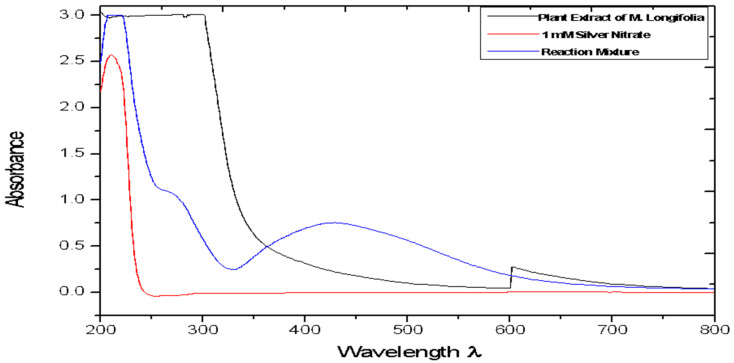
UV-Vis spectra of reaction mixture and control recorded after 5 min of exposure to sunlight.

The UV visible spectroscopy measurements were used to confirm the formation of silver nanoparticles. The spectra of the reaction mixture after 5 min of exposure to sunlight are shown in Figure 1. It is well known that the silver nanoparticles exhibit absorption at around wavelength 440 nm. This absorption of the spectra occurs as electrons on the metal surface undergo oscillations when excited by light at specific wavelengths. In the present study, the reaction mixture exhibits a unique peak at around 440 nm confirming the formation of silver nanoparticles [14].

A plant is the best option for producing nanoparticles, since the methods are free of toxic chemicals; in addition, plants also produce natural capping agents [28]. A UV-vis spectrophotometer was used to monitor the formation and stability of silver nanoparticles in the colloidal solution. It is generally recognized that UV–Vis spectroscopy could be used to examine the size and shape-controlled nanoparticles in aqueous suspensions [19]. In the present study, it has been found that the leaves of *Madhuca longifolia*, a traditional medicinal plant, have the potential to reduce silver nitrate ions to silver nanoparticles. The light brown color of the aqueous extract of *Madhuca longifolia* leaves was changed to reddish brown after 5 min of exposure to sunlight. The intensity of the color increased with the incubation time [24]. Maximum absorbance at 440 nm was observed, which is characteristic of silver nanoparticles [14].

### 3.2. FTIR-(Fourier Transforms Infrared Spectroscopy)

**Figure 2:**
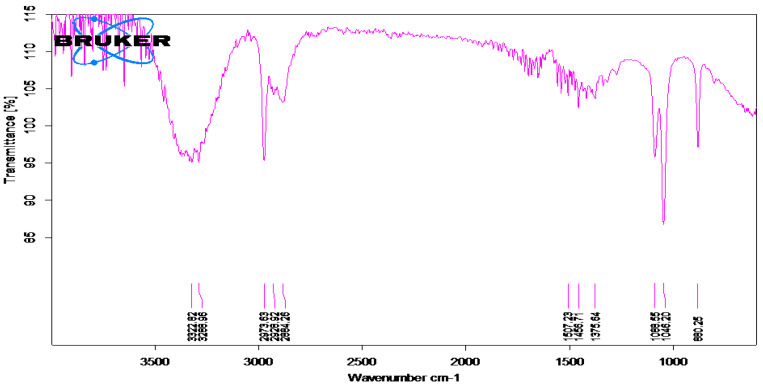
FTIR spectrum of *Madhuca longifolia* leaf extract.

**Figure 3:**
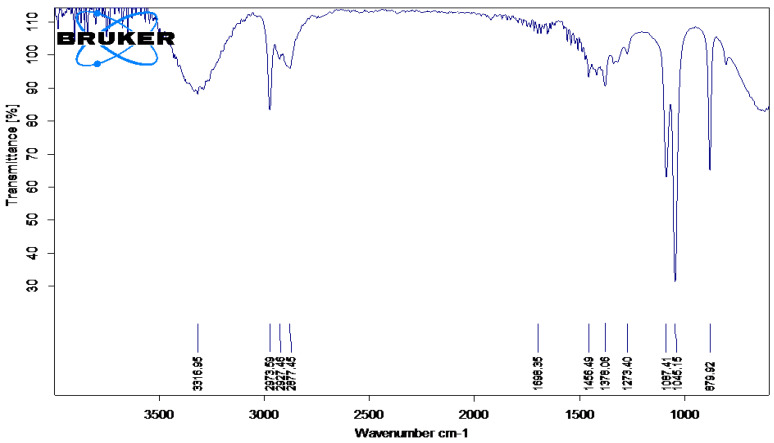
FTIR spectrum of AgNps synthesized by *Madhuca longifolia* leaf extract.

The IR spectra of plant extract of Madhuca longifolia, and of the reaction mixture exposed to sunlight, are shown in Figure 2 and Figure 3, respectively. Peak assignments were used to identify the functional groups [29]. 

The IR spectral analysis of the plant extract and reaction mixture reveals the presence of carbohydrates, glycosides and flavonoids. These biomolecules might have played a major role in capping and reducing agents during the formation of silver nanoparticles [30]. The IR spectra for both (plant extract and reaction mixture) showed an intense peak at around 3200 cm^−1^ that can be assigned to phenolic O-H stretching. The peak at 3000 cm^−1^ can be assigned to N-H stretching, 1610 cm^−1^ can be assigned to C=C aromatic stretching, while the peak at 1500 cm^−1^ may be because of N-O stretching. The intense peak at 1112 cm^−1^ can be assigned to C=O stretching (s) and 1000 cm^−1^ assigned to C=C bending of the molecules, probably flavonoids, glycosides, carbohydrates, tannins present in the extract and reaction mixture [22].

FT-IR (Fourier-Transform InfraRed spectroscopy) analysis of an infrared spectrum represents a fingerprint of a sample with absorption peaks which correspond to the frequencies of vibrations between the bonds of the atoms making up the material. Because each different material is a unique combination of atoms, no two compounds produce the same infrared spectrum. Therefore, infrared spectroscopy can result in an identification (qualitative analysis) of every different kind of material [31]. The FTIR spectrophotometer was used to identify the functional groups present in phytoconstituents responsible for reducing and capping AgNPs [32]. FTIR spectrum analysis in this study detected several absorption peaks. It exhibited N–H stretching vibrations, indicating strong hydrogen bonding, and C=O extension vibrations attributed to carboxylic acids, ketones, and aldehydes, which were linked to the silver ions reduction [14]. FTIR study indicates that the carboxyl (-C=O), hydroxyl (-OH), and amine (N-H) groups present in leaf extract are involved in the reduction of silver ions [33].

### 3.3. TEM (Transmission Electron Microscopy)

**Figure 4:**
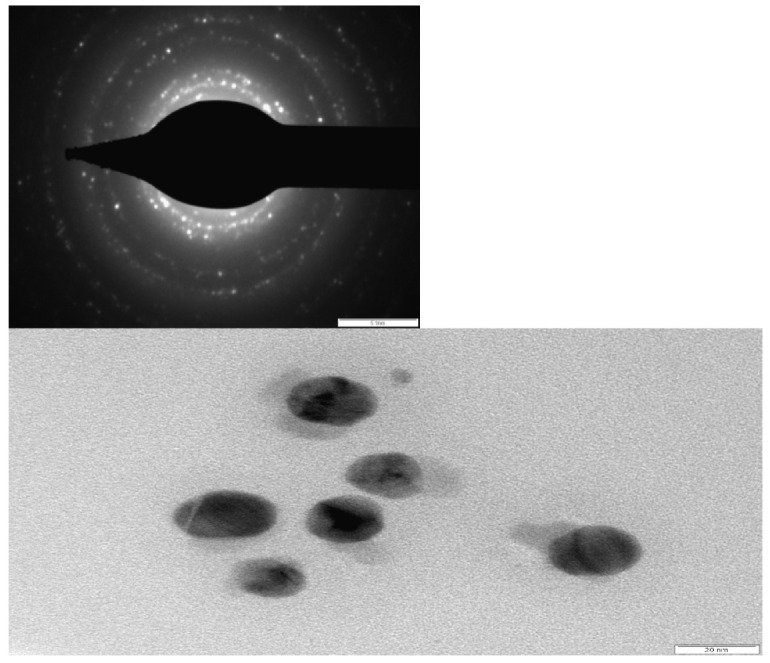
TEM images of AgNps synthesized using dried leaf extract *M. longifolia* insight Selected Area Electron Diffraction (SAED) pattern of AgNPs.

The shape and size of the resultant particles were elucidated with the help of TEM Figure 4. Aliquots of Ag nanoparticle solution were placed on a carbon-coated copper grid and allowed to dry under ambient conditions and TEM images were recorded. The TEM micrographs suggest that the particles are mostly spherical and are of a uniform size of around 20 nm. The SAED pattern indicates the crystalline nature of silver nanoparticles. Analysis of leaf-mediated synthesis of silver nanoparticles by TEM images proved that the size of AgNPs is in the range of nanoscale, almost spherically shaped, and that they have a mean diameter of 20 nm. Most of the nanoparticles had smooth edges and were circular. In the TEM image, the AgNPs were in physical contact but scattered by an adequately uniform distance between particles [34].

### 3.4. X-ray Diffraction Analysis (XRD)

**Figure 5:**
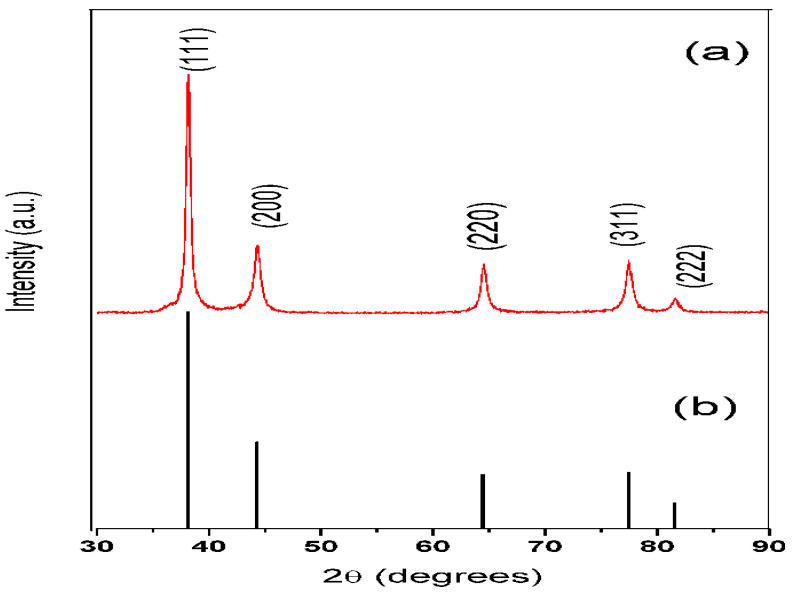
(**a**) XRD pattern for synthesized silver nanoparticles (**b**) Pattern from JCPDS file No. 04-078.

The synthesized silver nanoparticles purified by repeated centrifugation and washing were dried and analyzed on an X-ray diffractometer. X-ray powder diffraction is a rapid analytical technique that provides information on unit cell dimensions. Figure 5 shows the XRD pattern of silver nanoparticles with characteristic peaks observed at 2 θ values of 38.11º, 44.27º, 64.42º, 77.47º, and 82.0° that can be assigned to planes of (111), (200), (220), (311) and (222), respectively (JCPDS No. 04–0783) [22,23,24].

### 3.5. DLS (Dynamic Light Scattering)

**Figure 6:**
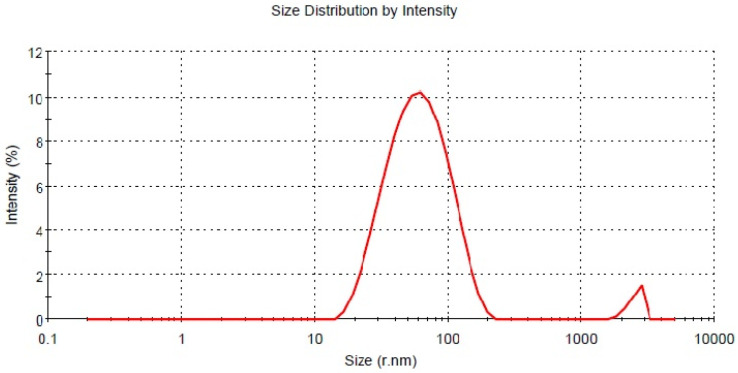
Size distribution of silver nanoparticles.

**Figure 7:**
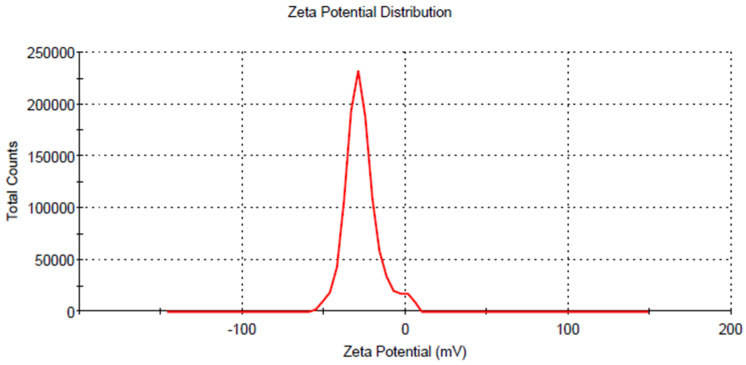
Zeta potential for silver nanoparticles.

The average hydrodynamic radius of AgNps synthesized using leaf extract of *M. longifolia* was found to be 45.30 nm Figure 6, with an asymmetric distribution between 13 to 200 nm. However, the TEM micrograph suggests that the particle size is around 20 nm. This disparity may be because all particles were taken into account for the calculation of hydrodynamic radius in DLS. The DLS data suggest that the particles are polydisperse. The Zeta potential of AgNps determined in water as a dispersant was −27.0 mV Figure 7. The high negative charge constitutes a repulsive barrier that physically separates the nanoparticles, avoiding aggregation [35].

A nanoparticle’s zeta potential is a key indicator for its stability in aqueous solution. For *M. longifolia* silver nanoparticles zeta potential measured was found to be −28 mV with a peak area of 100% intensity. These values indicate that the synthesized silver nanoparticles have excellent stabilization [36].

### 3.6. Phytochemical Screening of Leaf Extracts (Qualitative Screening)

To get an insight into the chemical constituent present in the leaves of *M. longifolia*, qualitative phytochemical analysis was carried out using standard procedures as described by Harborne (1973) [37]. The leaf extract of *Madhuca longifolia* shows positive tests for the bioactive compounds such as alkaloids, saponin, phytosterols, phenol, tannins, flavonoids, terpenoids, and ascorbic acid. Various phytochemical compounds detected are known to have medicinal importance. 

### 3.7. Antibacterial Activity

The silver nanoparticles exhibited activity against the gram-negative bacteria *E. coli*. All the concentration studies showed a zone of inhibition. The zone of inhibition was concentration dependent (Table 1). The maximum antibacterial activity against *E. coli* with a zone of inhibition was 16 mm at 200 μg/ mL of silver nanoparticles; furthermore, there was no increase in the zone of inhibition with an increase in the concentration of silver nanoparticles [38].

The antimicrobial efficacy of the nanoparticles depends on the shapes and size of the nanoparticles. The mechanism of action of silver nanoparticles on bacterial cells is well studied. It has been reported that the silver ions, after their entry into the bacterial cell, interact with sulfur and phosphorus present in DNA; the nanoparticles can act on these soft bases and destroy the DNA, which leads to cell death [39]. It also hampers DNA replication and terminates the growth of microbes [40]. In the present study, AgNPs showed maximum activity against *E. coli* with a zone of inhibition of 20 mm, which is comparable with standard antibiotics.

### 3.8. In-Vitro Anti-Inflammatory Activity

The in vitro anti-inflammatory activity was studied using protein denaturation and the (human red blood cell) HRBC membrane hemolysis method. Table 2 shows the percent inhibition of protein denaturation for different concentrations of silver nanoparticles and sodium diclofenac. The silver nanoparticles exhibited good anti-inflammatory properties that are comparable with standard sodium diclofenac. Protein denaturation is a process by which proteins lose their tertiary and secondary structures by application of external stress compounds such as strong acid, base, concentrated inorganic-organic salt, or heat. Protein denaturation is a well-documented causative of inflammation. During this process, the proteins lose their quaternary structure, thereby inducing aggregation, which activates deleterious inflammatory signals [41]. Inhibition of albumin denaturation by silver nanoparticles in the present study indicated anti-inflammatory activity [15].

The stabilization of the HRBC membrane by hypotonicity-induced and heat-induced membrane lysis was studied to establish the mechanism of anti-inflammatory action of silver nanoparticles synthesized using *Madhuca longifolia*. The percent inhibition of hemolysis studied using heat and hypotonicity showed an increasing trend, with an increase in the concentration of silver nanoparticles (Table 3). At 500 μg/mL concentration of AgNps, it was comparable with standard sodium diclofenac 100 μg/mL concentration in both cases. The results indicate a reduction in inflammation that can be due to the presence of active-principle components capped on silver nanoparticles. There was a significant increase in absorbance due to the treatment of *Madhuca longifolia* AgNPs in comparison to that with sodium diclofenac. For all the data, the values are significant at the *P* < 0.001 level. Thus, the results reveal that sodium diclofenac has hepatoprotective potential.

The membrane stabilization effect as a measure of anti-inflammatory activity was used in the present study. On exposure of RBCs to the hypotonic solution, excessive accumulation of fluid within the cell resulted in membrane rupture followed by hemolysis [25].

It is well established that protein denaturation inhibitors’ agents function by suppressing different types of inflammatory mediators involved in the inflammation process [42]. Denaturation of proteins is a well-documented cause of inflammation; drugs such as Phenylbutazone, salicylic acid, sodium diclofenac, and flufenamic acid show dose-dependent ability against protein denaturation [43]. It has also been reported that various plant extracts and their isolated compounds showed good anti-inflammatory activity comparable to that of synthetic anti-inflammatory drugs [44]. 

One of the causes of rheumatoid arthritis is the denaturation of proteins, and inhibition denaturation is one of the in vitro tests used to screen anti-inflammatory drugs [45]. Those drugs that are available are known as nonsteroidal anti-inflammatory drugs (NSAIDs), which act by inhibiting the function of prostaglandin. Prostaglandin is an autocoid that is released extracellularly and triggers pain. Anti-inflammatory agents block this autacoid’s synthesis by either inhibiting the COX enzyme or protecting the lysosomal membrane from breakdown [46]. The majority of the anti-inflammatory drugs stabilize the plasma membrane of mammalian erythrocytes and thereby inhibit the heat-induced and hypotonicity-induced hemolysis [47].

The AgNPs at concentrations of 100 to 500 μg/ mL protected the human RBC membrane against lysis induced by hypotonic solution and heat during inflammation. Membrane stabilization leads to the prevention of leakage of serum protein and fluids into the tissues during a period of increased permeability caused by inflammatory mediators [48]. The AgNPs perhaps stabilized the red blood cell membranes by preventing the release of lytic enzymes and active mediators of inflammation. The RBC membrane was effective against hypotonicity-induced hemolysis, and hence it would be effective in the form of non-steroidal complex anti-inflammatory compounds in the control of inflammation [33].

#### Antioxidant Activity by TLC DPPH Method TLC Profiling

**Figure 8:**
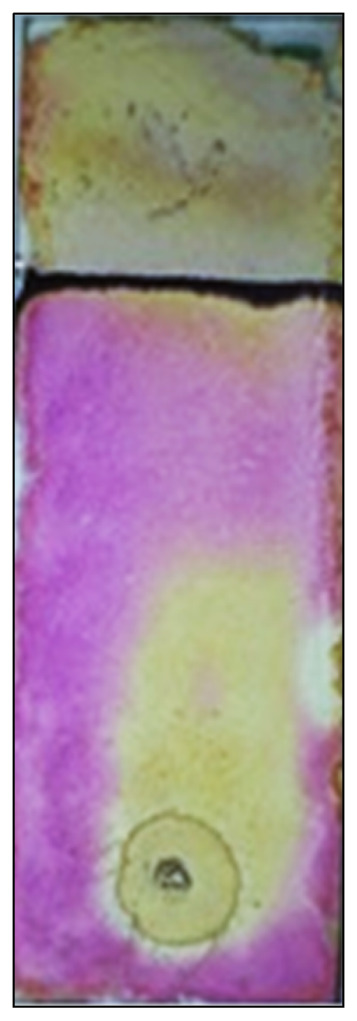
TLC-DPPH assay of *M. longifolia* leaf extract after spraying DPPH.

The dot blot study has shown an intense white spot on a light purple background (Figure 8) indicating the scavenging activity of silver nanoparticles. The spectrophotometric analysis of percent scavenging activity demonstrated by various concentrations of silver nanoparticles is shown in Table 4. All the concentrations have shown scavenging activity; however, it was highest with 500 μg/mL of AgNps.

In the present study, DPPH radical was used as a substrate to identify the free radical scavenging activity of AgNps. Among different concentrations of 100–500 μg/ mL the scavenging activity of AgNPs expressed the highest antioxidant activity causing 23.33, 24.33, and 29.66% inhibition of DPPH free radicals at 300, 400, and 500 μg/mL concentration, respectively. Ascorbic acid was used as positive control and its percent inhibition was 84% at 500 μg/mL concentration. The soluble compounds (flavanoids, glycosides, phenolic compounds etc) present in the methanolic leaf extract serve as a significant indicator of its potential antioxidant activity. Results suggests that the *Madhuca longifolia* possesses potent antioxidant properties by reducing reactive oxygen species as well as having the capability to donate hydrogen atoms. There was significant variation in DPPH scavenging activity among different concentrations, *P* = 0.05. In comparison to ascorbic acid, the DPPH scavenging activity significantly decreased under the influence of various concentrations.

DPPH is a stable free radical that accepts an electron or hydrogen radical to become a stable diamagnetic molecule. The decrease in the absorbance of DPPH radical is caused by antioxidants, because the reaction between antioxidant molecules and radicals progresses, which results in the scavenging of the radical by hydrogen donation. It is visually noticeable as a change in color from purple to yellow. Hence, DPPH is usually used as a substrate to evaluate the antioxidative activity of antioxidants. From the results, it can be assumed that the silver nanoparticle synthesized using *Madhuca longifolia* possesses hydrogen-donating capabilities and acts as an antioxidant [49].

### 3.9. Anticancer Activity

The silver nanoparticles synthesized using *M. longifolia* leaf extract showed more significant anticancer activity (Table 5) compared to the positive control group, i.e., Adriamycin GI50 = Growth inhibition of 50% (GI550) calculated from [(Ti-Tz)/(C-Tz)] × 100 = 50, drug concentration resulting in a 50% reduction in the net protein increase. TGI = Drug concentration resulting in total growth inhibition (TGI) will be calculated from Ti = Tz LC50 = the percentage of drug concentration at the end of the drug treatment, indicating a net loss of 50% cells following treatment, which is calculated from [(Ti-Tz)/Tz] × 100 = −50.

## 4. Conclusions

Synthesis of nanoparticles using biological agents is eco-friendly, low cost, and can be produced at room temperature. The present study has shown that phytochemicals from *Madhuca longifolia* leaf extracts have acted as both reducing and stabilizing agents. The silver nanoparticles were synthesized rapidly when exposed to sunlight for 5 min. They are spherical and uniform, capped with phytochemicals present in the leaf extract of *Madhuca longifolia,* as confirmed by various characterization techniques. In this process, the main phenolic compounds, triterpenoids, glycosides present in the leaf extract may be related with the chemical reduction of silver ions and thereby form nanoparticles with an average diameter on 20 nm. The AgNps are potent in inhibiting *E. coli* bacteria and showed significant antioxidant, anticancer, and anti-inflammatory activity. The outcome of this research confirms that *M. longifolia* extract is able to form silver nanoparticles and exhibits potent biological activities.

## Figures and Tables

**Table 1:** Antimicrobial activity of *M. longifolia* Leaf Nanoparticles.

Name of Organism *E. coli*	*Madhuca longifola* AgNPs concentration (µg/mL) wt/v					STANDARD (ciprofloxacin zone of inhibition in mm) (5 µg/disc)
	50	100	150	200	250	
Zone of inhibition in mm including disk (AgNPs)	10	11	13	16	20	20
Zone of inhibition in mm including disk (Crude)	−	10	13	13	14	22

**Table 2:** Anti-inflammatory activity of silver nanoparticles studied using BSA denaturation method.

Concentration of AgNps (μg/mL) and sodium diclofenac (μg/mL)	Percent inhibition	
	Sodium Diclofenac	AgNps
100	21.48 ± 0.51	20.60 ± 0.22
200	28.17 ± 0.78	23.80 ± 0.41
300	33.28 ± 1.12	33.17 ± 1.35
400	42.16 ± 0.31	39.48 ± 0.26
500	56.68 ± 0.92	53.15 ± 0.87

**Table 3:** Effect of different concentrations of AgNPs and standard (sodium diclofenac) on HRBC membrane hemolysis of erythrocyte using heat-induced and hypotonicity studies.

Treatment(s)	Concentration (μg/mL)	Absorbance at 560 nm		%Inhibition of hemolysis	
		For Heat-induced studies	For Hypotonicity induced studies	For Heat-induced studies	For Hypotonicity induced studies
Control	−	0.32	0.33	−	−
*M.longifolia* AgNps	100	0.39	0.42	21.87	27.27
	200	0.24	0.40	25	21.21
	300	0.20 *	0.24 NS	37.5	27.27
	400	0.18 *	0.21 NS	43.75	36.36
	500	016 NS	0.14 NS	50	57.57
Sodium Diclofenac	100	0.08	0.13	75	60.60

SE = of six individual observations, * values are significant at *P* < 0.001, NS = non-significant.

**Table 4:** Percent scavenging activity showed by AgNPs and ascorbic acid.

Concentration (μg/mL)	DPPH% Scavenging activity		
	AgNps	Crud extract of leaf	Ascorbic acid
100	7.6 *	7.0	78
200	14.33 *	10.89	80
300	23.33 *	16.00	82
400	24.33 *	20.45	83
500	29.66	22.18	84

SE= of six individual observations, * values are significant at *P* < 0.005 and *P* < 0.001.

**Table 5:** Anticancer activities of silver nanoparticles and control (ADR).

**Samples**	**Human Cervical Cancer Cell Line SiHa**			
	**Drug Concentration (μg/mL)**			
	10	20	40	80
Silver nanoparticles	96.4	105.6	107.9	133.5
ADR	−51.2	−27.6	−34.5	−23.1
	**Human Breast Cancer Cell** **Line MDA-MB-231**			
Silver nanoparticles	104.5	99.8	82.8	53.7
ADR	−48.0	−55.6	−56.7	−49.9
**LC 50**	**TGI**		**GI50 studies**	
**Human Cervical Cancer Cell** **Line SiHa**				
	LC 50	TGI	GI50	
Silver nanoparticles	>100	>100	>80	
ADR	52.5	<10	<10	
**Human Breast Cancer Cell** **Line MDA-MB-231**				
Silver nanoparticles	>100	>100	>80	
ADR	44.1	<10	<10	

LC50 = Concentration of drug causing 50% cell kill; GI50 = Concentration of drug causing 50% inhibition of cell growth; TGI = Concentration of drug causing total inhibition of cell growth; ADR = Adriamycin, Positive control compound.

## Data Availability

All data used to support the findings of this study are included within the article.
